# The Role of Chemokines in Promoting Colorectal Cancer Invasion/Metastasis

**DOI:** 10.3390/ijms17050643

**Published:** 2016-04-28

**Authors:** Yoshiro Itatani, Kenji Kawada, Susumu Inamoto, Takamasa Yamamoto, Ryotaro Ogawa, Makoto Mark Taketo, Yoshiharu Sakai

**Affiliations:** 1 Department of Surgery, Graduate School of Medicine, Kyoto University, Kyoto 606-8507, Japan; itatani@kuhp.kyoto-u.ac.jp (Y.I.); sinamoto@kuhp.kyoto-u.ac.jp (S.I.); masayama@kuhp.kyoto-u.ac.jp (T.Y.); ogawaryo@kuhp.kyoto-u.ac.jp (R.O.); taketo@mfour.med.kyoto-u.ac.jp (M.M.T.); ysakai@kuhp.kyoto-u.ac.jp (Y.S.); 2 Moores Cancer Center, University of California, San Diego, CA 92093, USA; 3 Department of Pharmacology, Graduate School of Medicine, Kyoto University, Kyoto 606-8507, Japan

**Keywords:** colon cancer, tumor microenvironment, myeloid cells, cancer immunity

## Abstract

Colorectal cancer (CRC) is one of the leading causes of cancer-related death worldwide. Although most of the primary CRC can be removed by surgical resection, advanced tumors sometimes show recurrences in distant organs such as the liver, lung, lymph node, bone or peritoneum even after complete resection of the primary tumors. In these advanced and metastatic CRC, it is the tumor-stroma interaction in the tumor microenvironment that often promotes cancer invasion and/or metastasis through chemokine signaling. The tumor microenvironment contains numerous host cells that may suppress or promote cancer aggressiveness. Several types of host-derived myeloid cells reside in the tumor microenvironment, and the recruitment of them is under the control of chemokine signaling. In this review, we focus on the functions of chemokine signaling that may affect tumor immunity by recruiting several types of bone marrow-derived cells (BMDC) to the tumor microenvironment of CRC.

## 1. Introduction

Although primary colorectal cancer (CRC) can be cured by surgical resection if without metastases, the patient survival rate worsens once it metastasizes to vital organs such as the liver or lungs. Primary CRC develops progressively through accumulation of genetic mutations in such genes as *APC*, *KRAS*, *p53*, *SMAD4* and *PTEN*, and epigenetic silencing of tumor suppressor genes [1,2]. In addition to these genetic and epigenetic alterations, there is mounting evidence that CRC accumulates invasive and metastatic capacities through tumor-stroma interactions, the so-called tumor microenvironment. Moreover, several further reports indicate that the tumor microenvironment not only promotes cancer invasion and metastasis but also provides resistance to chemotherapy [3]. Therefore, it is imperative to understand the tumor microenvironment thoroughly in order to achieve a comprehensive therapy, especially against advanced and metastatic CRC.

Chemokines are small peptides that are structurally and functionally similar to growth factors, binding to G-protein-coupled receptors to induce chemoattraction, inflammation and/or angiogenesis. They are also one of the key players that promote cancer cell metastasis in some types of cancers [4]. The tropism of metastatic cancer cells for specific organs can be aided by interactions between chemokine receptors on cancer cells and their respective ligands in the target organs. The role of chemokine receptors in metastatic organ selectivity was first reported for CXCR4, a receptor that stimulates metastasis of breast cancer and melanoma cells to the lung and lymph node where its ligand, CXCL12 (also known as SDF-1: stromal cell-derived factor 1), is abundant [5]. Furthermore, recent studies have suggested that chemokines are produced by cancer cells, leading to the recruitment of host cells into the tumor microenvironment. Several types of host cells interact with tumor epithelial cells in the tumor microenvironment and promote invasion and metastasis [6]. For example, cancer-associated fibroblasts (CAF) stimulate the growth of breast cancer cells through secretion of CXCL12 [7]. Mesenchymal stem cells (MSC) also play a role in breast cancer metastasis through secretion of CCL5 [8]. Recently, it has become increasingly evident that bone marrow-derived cells (BMDC) contribute to malignant progression has been increasingly evident [6,9,10,11,12]. Various mouse models have shown that BMDC play important roles in tumor progression: tumor-associated macrophages (TAM), myeloid-derived suppressor cells (MDSC) and tumor-associated neutrophils (TAN). However, it remains to be determined whether similar mechanisms are involved in humans. The best-characterized among them, TAM, are divided into two populations: M2 TAM that are pro-tumorigenic and M1 TAM that are anti-tumorigenic. MDSC constitute a heterogeneous population of immature myeloid cells that increase in number in cancer, inflammation and infection [13]. Human MDSC lack expression of conventional cellular markers for T cells, B cells, natural killer (NK) cells and macrophages, whereas they are positive for the common myeloid cell markers CD33 and CD11b, while negative for the human leukocyte antigen D-related (HLA-DR). MDSC can inhibit T cell reaction, antibody production and cytotoxic T lymphocyte (CTL) induction. They are comprised of two subpopulations; monocytic-MDSC (HLA-DR^−^, CD11b^+^, CD33^+^ and CD14^+^) and granulocytic-MDSC (HLA-DR^−^, CD11b^+^, CD33^+^ and CD15^+^) [14]. TAN also have differential states of activation and differentiation as similarly defined with M1 and M2 TAM: N1 TAN are anti-tumorigenic, whereas N2 TAN display pro-tumorigenic properties [15,16]. Several studies show that an elevated neutrophil count in the peripheral blood (*i.e.*, a high neutrophil-to-lymphocyte ratio [NLR]) is associated with poor clinical outcomes in many types of cancer including CRC [17], emphasizing the importance and relevance of neutrophils in cancer biology.

Tumor tissues consist of both cancer epithelial cells and normal stromal cells. In the tumor stroma, there are several types of host structures as extracellular matrix and cells including vascular endothelial cells, fibrous tissue cells (e.g., CAF and MSC), and immune cells such as NK cells, dendritic cells (DC), eosinophils, basophils, mast cells, regulatory T cells (T-reg), tumor infiltrating lymphocytes (TIL), TAM, TAN and MDSC [18]. Cancer cells induce inflammation through secretion of several cytokines and chemokines in their invasive process. Dysregulation of tissue homeostasis can cause to extensive cytokine production, a cytokine burst, allowing cancer cells to survive by hijacking the normal immune system. Some of them have been elucidated to suppress anti-tumor immunity and promote tumor expansion in the surrounding stromal tissues.

In this review, we will discuss the roles of several types of BMDC in constructing the metastatic microenvironment for CRC through chemokine signaling. Accordingly, we list the chemokine ligand-receptor interactions that are reported so far to be involved in CRC progression by affecting myeloid cells in the tumor microenvironment (Table 1).

## 2. CXCL12 and CXCR4

The CXCL12-CXCR4 axis is one of the representative chemokine signaling involved in cancer metastasis [5]. Namely, breast cancer cells that express CXCR4 frequently metastasize to the lung, liver, lymph node and bone marrow where CXCL12 is expressed at high levels. In the liver, one of the most frequent sites of CRC metastasis, CXCL12 is secreted from the endothelial cells, Kupffer cells and α-smooth muscle actin (α-SMA)-positive myofibroblasts [29,30]. Fibroblasts often constitute the majority of the stromal cells, and CAF extracted from invasive human breast cancer promotes tumor growth and angiogenesis through CXCL12 secretion in a mouse model [7]. Autocrine tumor growth factor beta (TGF-β) and CXCL12 signaling loops initiate and maintain the differentiation of fibroblasts into myofibroblasts and the concurrent tumor-promoting phenotype [47]. In CRC, it has been shown in clinical studies that CXCR4 expression correlates with tumor recurrence, poor survival and liver metastasis [22,23,25,26,27,28]. Expression of CXCR4 in CRC cell lines was also upregulated under hypoxia by hypoxia-inducible factor 1-alpha (HIF-1α) activity [48]. Its ligand, CXCL12, is also secreted by CRC cells, but the significance of CXCL12 expression by cancer cells remains controversial so far. Namely, CXCL12 expression in CRC cells appears to be bidirectional: tumor promotive [31] or tumor suppressive [49]. Even in the latter case, CXCR4 and CXCL12 were reported to be reciprocally expressed in CRC [50], indicating that CRC cells always show high-level expression of either CXCL12 or CXCR4, but not both. As a result, we have come to the consensus that pharmacologic inhibition of the CXCL12–CXCR4 axis may suppress CRC metastasis and prolong the survival period [51,52].

Activation of the CXCL12–CXCR4 axis recruits not only CXCR4^+^ cancer cells but also CXCR4^+^ BMDC that promote angiogenesis in a vascular endothelial growth factor (VEGF)-dependent [53] or -independent [54] manner. The contribution by myeloid BMDC in cancer metastasis was first reported as a “pre-metastatic niche” mouse model where CD11b^+^ VEGFR1^+^ BMDC accumulate at a niche of the lung before the arrival of cancer cells [55]. In this model, CXCL12 secreted by BMDC attract CXCR4^+^ tumor cells to the pre-metastatic niche through the CXCL12–CXCR4 axis, aiding lung metastasis. It was also reported that VEGFR1 and CXCR4 independently caused a pro-migratory effect on BMDC through the VEGF–VEGFR1 and CXCL12–CXCR4 pathways, respectively [54]. Recently, a humanized antibody targeting CXCL12 has been established [56]. Treatment with this antibody dramatically inhibits distant metastases in multiple xenografts and breast cancer orthotopic models, which may also bring a significant benefit for the treatment of CRC as well.

## 3. CXCL9/10 and CXCR3

CXCR3 may provide details of one of the attractive explanations how cancer cells metastasize to their draining lymph nodes. We earlier reported that CXCR3 expressed on melanoma and CRC cells promoted their metastases to the draining lymph nodes where CXCL9/10, ligands of CXCR3, are expressed at high levels [22,23,57,58]. We also found that CRC patients with CXCR3^+^ tumors showed significantly shorter survival rates than those without CXCR3, and that CRC patients with tumors double-positive for both CXCR3 and CXCR4 had a significantly poorer prognosis than those with tumors positive only for CXCR4 or those that were double-negative [22,58]. It was also reported that CXCR3 expression was up-regulated in metastatic CRCs compared to their primary counterparts, and that activation of the CXCL10–CXCR3 axis significantly up-regulated invasion-related properties in CRC [59]. In addition to CRC, CXCR3 was also reported to be correlated with a poor prognosis in breast cancer and melanoma [60,61]. Notably, systemic administration of a small molecular inhibitor of CXCR3 (AMG487) inhibited lung metastasis of CRC as well as breast cancer in murine models [62,63]. In host-targeted investigations, it has been reported recently that deletion of CXCR3 in all host cells significantly decreased lung metastasis of breast cancer by decreased expression of IL-4, IL-10, inducible nitric oxide synthase (iNOs) and arginase-1 (Arg1) in myeloid cells with an increased T cell response [64].

CXCL10, ligand for CXCR3, is also secreted by CRC cells, which recruits CXCR3^+^ helper T lymphocytes type 1 (Th1) at the invasion front of CRC and contributes to anti-tumor immunity [24]. Expression of CXCL10 in stage II and/or III CRC was reported to be an independent good prognostic factor regarding recurrence [65,66], whereas another recent study shows that co-expression of CXCL10 and CXCR3 in CRC is a predictor of metastatic recurrence and poor prognosis [67]. The serum CXCL10 level was reported to be associated with a poor prognosis for CRC patients, as well as a high malignant status such as high pathological T stage, vascular invasion and distant metastasis [68]. Further comprehensive studies are awaited to assess the significance of CXCL10 expression in CRC.

## 4. CXCL1/2/5/8 and CXCR2

CXC chemokines with an ELR (Glu-Leu-Arg) motif such as CXCL1, CXCL2, CXCL5 and CXCL8 bind to CXCR2, and promote angiogenesis. The pro-inflammatory lipid mediator prostaglandin E_2_ (PGE_2_) plays a critical role in promoting CRC growth through production of VEGF in the stroma, aiding tumor angiogenesis [69]. PGE_2_ also stimulates pro-angiogenic chemokine CXCL1 expression in human CRC cells [19]. CXCL1 released from CRC cells induces CXCR2^+^ endothelial cell migration, causing increased tumor microvessel formation. The VEGF-independent angiogenic function of PGE_2_-derived CXCL1 may explain the refractoriness of anti-VEGF treatment in CRC patients. Consistently, CXCL1 expression is up-regulated in human CRC specimens compared with the adjacent normal tissues [70]. CXCL1 also contributes to tumor cell transformation, growth and invasion; the knockdown of CXCL1 in CRC cells causes inhibition of cell viability, resulting in the prevention of tumor growth in mouse models [71]. By comparing gene signatures of breast cancer cell lines with weak or strong metastatic tropism for the lung, CXCL1 is one of the gene products that promote lung metastasis [72]. The CXCL1–CXCR2 axis is also important for the formation of the pre-metastatic niche of CRC liver metastasis, and TSU68, an anti-angiogenic receptor tyrosine kinase inhibitor, suppressed CXCL1 expression in the pre-metastatic liver, resulting in suppression of the homing of CXCR2^+^ neutrophils and subsequent liver metastasis in a mouse model [20]. These results strongly suggest that anti-CXCL1 treatment may become an important strategy when used alongside the conventional chemotherapy with or without anti-VEGF therapy that already is being used worldwide.

In a breast cancer model (PyMT/Tgfbr2^KO^ mammary carcinoma), a mutated TGF-β receptor causes recruitment of CD11b^+^ Gr-1^+^ MDSC at the tumor invasion front via the CXCL5–CXCR2 and CXCL12–CXCR4 axes, and contributes to tumor invasion and lung metastasis through metalloproteinase (MMP) secretion [73]. It was also reported that loss of TGF-β signaling promoted CXCL1 and CXCL5 secretion, which increased migration of CD11b^+^ Gr-1^+^ MDSC to the tumor tissues, and played indirect roles in increasing the number of T helper 17 (Th17) cells which have a pro-tumorigenic effect [74]. Given the implication of CD11b^+^ Gr-1^+^ MDSC in suppressing tumor immunosurveillance, angiogenesis and tumor invasion, it is possible that targeting the homing or function of MDSC becomes therapeutically effective. MDSC express chemokine receptors such as CXCR2 and CCR2 in several types of cancer [21,75]. In a mouse model of colitis-associated CRC, the levels of CXCR2 ligands were significantly elevated in inflamed colonic mucosa and tumors compared with in adjacent normal mucosa, and CXCR2^+^ MDSC accelerated tumor growth by inhibiting CD8^+^ T cell cytotoxicity activity [21]. Genetic depletion of *Cxcr2* (*Cxcr2* knockout mice) diminished massive infiltration of granulocytic-MDSC into the tumors and retained them in their circulatory system, reducing chronic colonic inflammation and colitis-associated tumorigenesis. In CRC patients, the number and proportion of circulating and tumor-infiltrating MDSC were markedly increased compared with healthy individuals, and these increases were closely correlated with a clinical stage and tumor metastasis [76]. Tumor signaling through programmed death 1 (PD-1) on T cells and expansion of MDSC are major mechanisms of tumor immune escape. Notably, prevention of CXCR2-mediated MDSC trafficking by anti-CXCR2 mAb therapy enhances anti-PD-1 efficacy in a mouse model of rhabdomyosarcoma, suggesting a translatable strategy to improve the efficacy of immune checkpoint blockade therapy by preventing trafficking of MDSC to the tumor site [77].

CXCL8 (IL-8) is the first-described angiogenic chemokine with an ELR motif, and is secreted by CRC cells exposed to pro-inflammatory cytokines such as TNF-α and IL-1α [78]. CXCL8 can be induced by hypoxia even in HIF-1-deficient CRC cells as a compensatory pathway of VEGF to preserve tumor angiogenesis, suggesting the potential for combination regimens that target both HIF-1 and CXCL8 [79]. CXCL8 was also reported to be associated with metastatic potential and a resistance to oxaliplatin [80]. In mice that express human CXCL8, it contributes to colon carcinogenesis by increased mobilization of CD11b^+^ Gr-1^+^ immature myeloid cells [81]. In *Cxcr2* knockout mice, lack of the CXCL8–CXCR2 axis in the tumor microenvironment prevented CRC growth and metastasis [82]. In addition, small molecule antagonists of CXCR1 and CXCR2 inhibited liver metastasis of CRC by decreasing tumor angiogenesis and inducing tumor cell apoptosis in a mouse model [83]. Increased serum CXCL8 concentrations have been associated with distant metastasis and advanced clinical stages of CRC [80].

## 5. CCL15 and CCR1

SMAD4 is a transcription factor that plays a pivotal role in TGF-β signaling, and is one of the tumor suppressors of CRC [84,85,86]. In a genetically-engineered *Apc^+/∆716^/Smad4^+/−^* compound knockout mice that develop invasive intestinal adenocarcinomas [87], mouse CCL9 (mCCL9) is secreted from the cancer epithelium, which recruits CCR1^+^ myeloid cells to promote tumor invasion [38]. In a mouse model of liver metastasis, mCCL9-expressing CRC cell lines recruit CCR1^+^ myeloid cells to expand metastatic foci in the liver [39], and four distinct types of myeloid cells are found to be recruited to the liver metastatic foci: CCR1^+^ neutrophils, eosinophils, monocytes and fibrocytes [41]. Using *Ccr1* knockout mice, another group has reported that CCR1 expression by both hematopoietic and nonhematopoietic cells promotes liver metastasis through myeloid cell accumulation and angiogenesis [88]. In addition to these mouse models, we have recently reported that SMAD4 binds directly to the promoter region of human *CCL15* gene (a human ortholog of mouse *Ccl9*) and negatively regulates its expression [40]. Using human clinical specimens, we have also showed that loss of SMAD4 promotes CCL15 expression from CRC cells to recruit CCR1^+^ myeloid cells, which facilitates primary tumor invasion and liver metastasis of CRC [40,42]. Most CCR1^+^ cells accumulating at the invasion front of primary CRC were of the MDSC phenotype (CD11b^+^, CD33^+^, and HLA-DR^−^) [42]. Importantly, patients with CCL15-expressing CRC in their liver metastases showed significantly shorter relapse-free survival (RFS) than those with CCL15-negative CRC [40]. Stage II and III patients with CCL15-positive primary CRC also tended to have shorter RFS than those with CCL15-negative CRC [42]. In addition, we have found that loss of SMAD4 promotes lung metastasis of CRC by accumulation of CCR1^+^ TANs through the CCL15–CCR1 axis, and that CCL15 expression in lung metastases is an independent predictor of a poorer prognosis in CRC patients (Yamamoto *et al.* unpublished data). Notably, it has been reported that activation of the CCL2–CCR2 axis prompts TAM to secrete another chemokine CCL3, which in turn activates its receptor CCR1 in TAM and promotes lung metastatic seeding in a breast cancer mouse model [89]. These results suggest that inhibition of CCR1 may have a therapeutic implication on metastatic progression of certain types of cancer, including CRC and breast cancer. Some CCR1 inhibitors have been used clinically for patients with rheumatoid arthritis, multiple sclerosis or chronic obstructive pulmonary disease in phase I/II clinical trials [90,91]. Therefore, it would be worthwhile to test such CCR1 inhibitors in preventing cancer metastasis, because they have been already cleared from safety concerns.

## 6. CCL2 and CCR2

TAM play a key role in the process of colonic tumorigenesis and CRC progression through production of cyclooxygenase-2 (COX-2) [92]. CCL2, also called monocyte chemotactic protein 1 (MCP-1), is released from CRC and involved in macrophage accumulation and COX-2 expression [93]. CCL2 expression in CRC cells and TAM accumulation are strongly correlated with advanced tumor stages [32] and a poor prognosis [33]. Expression of CCL2 was also reported as a predictive marker for liver metastasis of human CRC [33]. The CCL2–CCR2 axis-mediated macrophage recruitment promotes tumor growth, progression and metastases in breast and prostate cancers [94,95]. In a genetic mouse model of CRC, inactivation of *Ccl2* caused depletion of CCR2^+^ TAM at the tumor site and reciprocal accumulation of CTL, which prevented tumor progression [96]. In a mouse model of colitis-associated carcinogenesis with azoxymethane followed by repetitive administration of dextran sulfate sodium, depletion of *Ccr2* (*Ccr2* knockout mice) reduced macrophage infiltration and tumor formation in the colonic mucosa [97]. In an experimental model of malignant pleural effusion, administration of a neutralizing antibody against CCL2 could inhibit development of malignant pleural effusion of CRC [98]. In an orthotopic transplantation mouse model of pancreatic cancer, CCR2 inhibitors depleted inflammatory monocytes and macrophages, which resulted in decreased tumor growth and reduced metastasis [99]. As an example of the paradoxical effect of CCL2, it has been reported that interruption of CCL2 inhibition accelerates breast cancer metastasis through angiogenesis, and that anti-IL-6 treatment after interruption of anti-CCL2 therapy can prevent lung metastases [100].

In a mouse allograft model, CCR2 is also expressed on CD11b^+^ Gr1^mid^ myeloid cells, which promotes liver metastasis of CCL2-expressing CRC cells [34]. Some CD11b^+^ CCR2^+^ cells are also found within the liver metastatic foci obtained from CRC patients, suggesting essentially the same mechanism can be occurred in the establishment of CRC liver metastasis in humans. Regarding the role of CCR2 on MDSC, it has been reported that CCL2 affects CCR2^+^ granulocytic-MDSC accumulation and function in CRC carcinogenesis, and that CCL2 modulates T cell suppression of granulocytic-MDSC in a STAT3-mediated fashion [35].

While CCR2^+^ myeloid cells contribute to tumor cell metastasis, CCR2^+^ endothelium is activated by CRC-derived CCL2, which increases vascular permeability and cancer cell extravasation through the JAK2-Stat5 and p38MAPK pathways [36].

## 7. CCL5 and CCR5

MSC are pluripotent progenitor cells that contribute to the maintenance and regeneration of a variety of connective tissues [101]. Recent reports have revealed that the bone-marrow-derived MSC are recruited in large numbers to the stroma of developing tumors [102]. Breast cancer cells stimulate CCL5 secretion from MSC, which then acts in a paracrine fashion on the CCR5^+^ cancer cells to enhance their motility, invasion and metastasis [8]. In addition to the CCL5–CCR5 axis, hypoxia induces HIF-dependent CXCL10 secretion from MSC, which recruits CXCR3^+^ breast cancer cells and promotes lung metastasis through the CCL10–CXCR3 axis [103]. CXCR3 is also expressed on CRC as mentioned above, and therefore a similar mechanism may operate in CRC metastasis.

T cell infiltration is often found in CRC tissues and is associated with a good prognosis for CRC patients [104]. For example, memory and effector-type T cell infiltration in the stroma of CRC is correlated with negative lymph node metastases, negative distant metastases and negative status of vessels/perineural invasion, which leads to a prolonged overall survival (OS) and disease-free survival (DFS) [105]. Moreover, CD8^+^ CTL infiltration is also found in CRC tissues, which can raise anti-cancer immunity that prolongs patients’ survival [106]. Most of the CD8^+^ CTL and type-1 helper T cells (Th1) at the invasion front of CRC tissues express CCR5, and expression of its ligand CCL5 in the tumor microenvironment is observed predominantly in the stromal CD8^+^ T lymphocytes at the invasion front [24], which may accelerate anti-tumor immunity in an autocrine and/or paracrine manner. Confusingly, another report shows an opposite relationship between CCL5 and CCR5 in the CRC microenvironment. Namely, CCL5 released from CRC cells recruits CCR5^+^ T-reg, which leads to TGF-β-mediated apoptosis of CD8^+^ CTL and tumor growth by immune escape [37]. Although CCL5 can bind to some chemokine receptors such as CCR1, CCR3 and CCR5, it is unclear which particular receptor is responsible for the pharmacologic CCL5 neutralization that can suppress cancer progression in mouse xenograft models [107]. Because these reports on the CCL5–CCR5 axis in the CRC tumor microenvironment appear to be contradictory, further investigation is awaited to determine whether the CCL5–CCR5 axis plays a role in anti-tumor immunity or tumor-promotion.

## 8. CCL20 and CCR6

Accumulation of Th17 lymphocytes in CRC tissues causes poor DFS, while that of Th1 lymphocytes results in prolonged DFS [108]. IL-17 produced from Th17 lymphocytes recruits CD11b^+^ Gr1^+^ immature myeloid cells to the tumor site, which induces granulocyte colony-stimulating factor (G-CSF) to promote tumor resistance to anti-angiogenic therapy (VEGF inhibition) [109]. T-reg also negatively regulate anti-tumor immunity to facilitate tumor growth, and depletion of total CD4^+^ T cells improves tumor immunity with effective tumor rejection [110]. Both Th17 and T-reg express CCR6, which promotes the migration of these two T cell subsets to the inflammatory site [111]. Moreover, a subset of IL17^+^ T-reg is induced from memory CCR6^+^ T cells [112]. In CRC tissues, the main source of CCL20, a ligand for CCR6, is considered to be TAM, and CCL20 secreted from TAM recruits CCR6^+^ T-reg to the tumor site, which synergistically promotes tumor progression in mouse models [43]. In human clinical samples of primary and metastatic CRC, expression of CCR6 and CCL20 is increased compared with the normal mucosa [113,114]. Both normal colonic epithelial cells and CRC cells have been demonstrated to bear CCR6 [115]. CCR6 expression in the primary CRC is independently associated with the presence of synchronous liver metastasis [116]. The serum level of CCL20 is also reported to be an independent predictive factor for CRC liver metastasis [117]. Recently, it has been reported that additional knockout of *Ccr6* into *Apc^Min/+^* mice, a mouse model of familial adenomatous polyposis, diminishes the number and size of their intestinal adenomas, suggesting that the intestinal tumorigenesis driven by the CCL20–CCR6 interaction may be promoted by macrophages recruited into the intestine [118].

## 9. CCL19/21 and CCR7

An effective T-cell response for anti-tumor immunity results from the interaction between antigen-presenting DC and naïve T cells in lymphoid tissues [119]. CCR7 is mainly expressed on some subsets of CD8^+^ T lymphocytes, naïve T lymphocytes (CCR7^+^ CD45RA^+^) and central memory T lymphocytes (CCR7^+^ CD45RA^−^), which may differentiate into CTL to acquire an anti-tumor function [44]. Its ligand, CCL19, is expressed from DC that are stimulated by NK cells through tumor necrosis factor α (TNF-α) and interferon γ (IFN-γ) [45]. Expression of CCL19 is up-regulated in lymphoid tissues obtained from CRC patients when treated *ex vivo* with factors that can stimulate NK cell function [45]. Expression of CCL19 in human CRC specimens is suppressed when compared with that in normal intestine [120]. Expression of CCL21, another ligand for CCR7, is also decreased in CRC tissues [121]. Accordingly, a higher infiltration of CCR7^+^ T lymphocytes into CRC tissues may predict prolonged OS and progression-free survival (PFS) [122]. In contrast, some reports show that CCR7 expression on CRC cells themselves plays an important role in cancer progression. CCR7 expression at the invasion front is strongly correlated with lymph node metastases and decreased OS [123]. When stimulated by CCL21, CCR7 on cancer cells is proposed to regulate MMP-9 and promote lymph node metastasis [124]. However, another report shows exactly the opposite result that CCR7 expression on CRC cells prolongs the 3-year survival rate [27]. There are no *in vivo* studies of mouse models that show the effect of the CCL19/20–CCR7 blockade on the progression of CRC.

## 10. CCL24 and CCR3

CCL24 expression is shown to be strongly up-regulated in the biopsy specimens of CRC liver metastases, as well as in the primary CRC, compared with the adjacent normal tissues [125]. Cancer epithelial cells as well as some of CRC cell lines showed high expression levels of CCL24, and CCL24 production by CRC cell lines was modulated by Th1/Th2 cytokines. Although little is known about the interaction of CCL24 with its receptor CCR3 in the tumor microenvironment *in vivo*, it may be worth investigating this chemokine axis for its possible role in CRC invasion and metastasis.

## 11. CX3CL1 and CX3CR1

MSC are also reported to migrate to the CRC microenvironment and differentiate to CAF, which promotes cancer expansion and metastatic focus formation [126]. One of the attractive explanations why MSC accumulate to CRC may lie in the CX3CL1–CX3CR1 axis. Expression of CX3CR1 is up-regulated on MSC when cultured under a hypoxic condition [127]. When co-cultured with CRC cell line HT29 under hypoxia, the migration ability of MSC increased *in vitro*. In clinical CRC specimens, CX3CR1 is expressed on TAM, and CX3CR1 up-regulation in TAM is correlated with a poor prognosis [46]. Apart from the cell types expressing CX3CR1, *Cx3cr1* knockout mice exhibited lower occurrence of liver metastasis when CRC cells were injected into the liver as allografts. TAM in the *Cx3cr1* knockout mice express significantly less angiogenic markers such as CCR2, VEGFR2, and CXCR4 compared with wild-type mice, suggesting synergistic effects on macrophage accumulation between angiogenesis and the CX3CL1–CX3CR1 axis. These results suggest that a blockade of the CX3CL1–CX3CR1 axis may be an attractive therapeutic target for advanced CRC.

CX3CR1 is also expressed on NK cells and CTL as well as TAM [128]. Because CX3CR1 is expressed in a wide variety of cell types, it is controversial whether the CX3CL1–CX3CR1 axis is tumor-suppressive or tumor-promotive. CX3CL1 overexpression in CRC cell lines can reduce their metastatic potential in a mouse allograft model [129]. CRC patients with higher expression of CX3CL1 were also reported to show a better prognosis [130].

## 12. Conclusions

**Figure 1:**
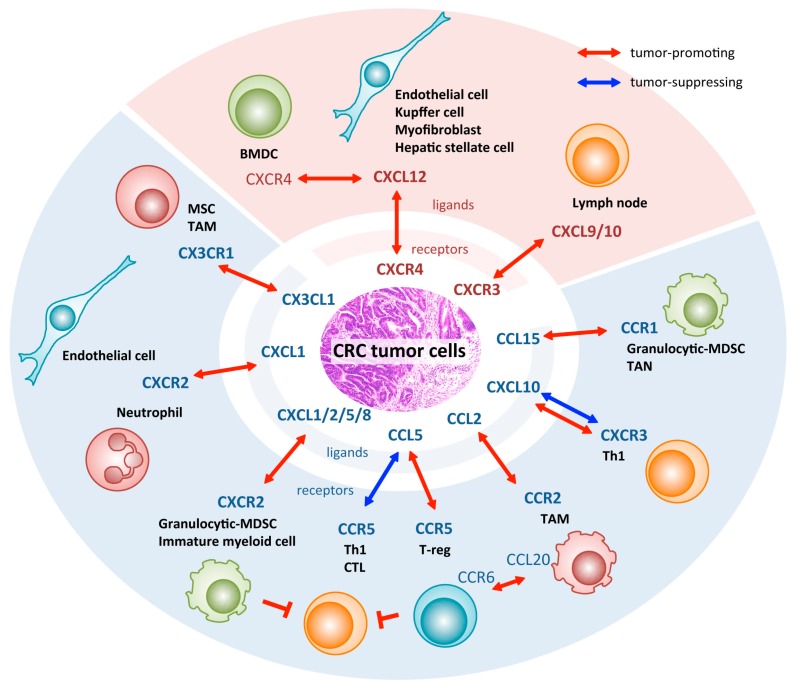
Chemokine signaling that mediates interactions between CRC cells and myeloid cells in the tumor microenvironment.

Tumor cells not only accumulate genetic mutations in themselves but also affect their surrounding cells in order to survive in the chaos of the tumor microenvironment. They utilize chemokines in their armory against the attack from their host. As described in this review, recent studies indicated that most of the listed chemokines are up-regulated when normal colonic mucosa becomes cancerous and the cancer becomes more malignant (Figure 1). There are several chemokine inhibitors that are used in mouse models *in vivo*, or even in some clinical studies for immune-related diseases. However, currently, there is no targeted therapy for chemokines to prevent CRC invasion and/or metastasis, as well as other types of cancer.

Recently, it was reported that mismatch-repair status of CRC predicted a clinical benefit with the anti-PD-1 immune checkpoint inhibitor [131]. Mismatch-repair pathway is one of the main causes of CRC [132], and the study’s results may show the potential advantage of immunotherapy in some subtypes of CRC. Because chemokine signaling is closely associated with tumor immunity, and tumor immunotherapy has reached a new milestone with the launch of anti-immune checkpoint therapy such as anti-PD-1 antibody, effective chemokine-targeted therapy may become within reach in a near future.

## Figures and Tables

**Table 1:** Chemokine signaling reported to be involved in colorectal cancer (CRC) progression through interaction with BMDCs.

Chemokine Signaling	Expressing Cell Type		Function	Reference
	Ligand	Receptor		
CXCL1–CXCR2	CRC	EC *	Angiogenesis	[19]
	Liver	CRC	Liver metastasis	[20]
	Inflamed colon	MDSC	Colitis-associated tumorigenesis	[21]
CXCL9/10–CXCR3	Lymph node	CRC	Lymph node metastasis	[22,23]
	CRC	Th1	Anti-tumor immunity	[24]
CXCL12–CXCR4	Liver	CRC	Liver metastasis	[23,25,26,27,28]
	EC *, myofibroblast	CRC	Liver metastasis	[29,30]
	CRC	ND ^†^	Shorten OS and RFS	[31]
CCL2–CCR2	CRC	TAM	Disease progression	[32,33]
	CRC	BMDC	Liver metastasis	[34]
	CRC	MDSC	Carcinogenesis	[35]
	CRC	EC, monocyte	Extravasation and metastasis	[36]
CCL5–CCR5	CTL	CTL, Th1	Anti-tumor immunity	[24]
	CRC	T-reg	Tumor growth	[37]
CCL15-CCR1	CRC	BMDC	Invasion and liver metastasis	[38,39,40]
	CRC	TAN	Liver metastasis	[41]
	CRC	MDSC	Invasion	[42]
CCL20–CCR6	TAM	T-reg, Th17	Tumor progression	[43]
CCL19–CCR7	DC ^¶^	CTL	Anti-tumor immunity	[44,45]
CX3CL1–CX3CR1	ND ^†^	TAM	Liver metastasis	[46]

* EC: endothelial cell; ^¶^ DC: dendritic cells; ^†^ ND: not described.

## Abbreviations

ARG1	arginase-1
α-SMA	α-smooth muscle actin
BMDC	bone marrow-derived cells
CAF	cancer-associated fibroblasts
COX-2	cyclooxygenase-2
CRC	colorectal cancer
CTL	cytotoxic T lymphocytes
DC	dendritic cells
DFS	disease-free survival
EC	endothelial cells
ECM	extracellular matrix
FAP	familial adenomatous polyposis
G-CSF	granulocyte colony-stimulating factor
HIF-1α	Hypoxia-inducible factor 1-alpha
HSC	hepatic stellate cells
IFNγ	interferon γ
iNOS	inducible nitric oxide synthase
LOH	loss of heterozygosity
MCP-1	monocyte chemotactic protein 1
MDSC	myeloid-derived suppressor cells
MMP	matrix metalloproteinase
MSC	mesenchymal stem cells
NK	natural killer
NLR	neutrophil-to-lymphocyte ratio
OS	overall survival
PD-1	programmed death 1
PFS	progression-free survival
PGE_2_	proinflammatory mediator prostaglandin E_2_
RFS	relapse-free survival
SDF-1	stromal cell-derived factor 1
TAM	tumor-associated macrophages
TAN	tumor-associated neutrophils
TIL	tumor infiltrating lymphocytes
TNF-α	tumor necrosis factor α
TGF-β	transforming growth factor-beta
Th1	type 1 helper T cells
Th17	T helper 17
T-reg	regulatory T cells
VEGF	vascular endothelial growth factor

## References

[1] Vogelstein B., Kinzler K.W. (2004). Cancer genes and the pathways they control. Nat. Med..

[2] Fearon E.R., Vogelstein B. (1990). A genetic model for colorectal tumorigenesis. Cell.

[3] Meads M.B., Gatenby R.A., Dalton W.S. (2009). Environment-mediated drug resistance: A major contributor to minimal residual disease. Nat. Rev. Cancer.

[4] Balkwill F. (2004). Cancer and the chemokine network. Nat. Rev. Cancer.

[5] Müller A., Homey B., Soto H., Ge N., Catron D., Buchanan M.E., McClanahan T., Murphy E., Yuan W., Wagner S.N. (2001). Involvement of chemokine receptors in breast cancer metastasis. Nature.

[6] Joyce J.A., Pollard J.W. (2009). Microenvironmental regulation of metastasis. Nat. Rev. Cancer.

[7] Orimo A., Gupta P.B., Sgroi D.C., Arenzana-Seisdedos F., Delaunay T., Naeem R., Carey V.J., Richardson A.L., Weinberg R.A. (2005). Stromal fibroblasts present in invasive human breast carcinomas promote tumor growth and angiogenesis through elevated SDF-1/CXCL12 secretion. Cell.

[8] Karnoub A.E., Dash A.B., Vo A.P., Sullivan A., Brooks M.W., Bell G.W., Richardson A.L., Polyak K., Tubo R., Weinerg R.A. (2007). Mesenchymal stem cells within tumour stroma promote breast cancer metastasis. Nature.

[9] Gao D., Mittal V. (2009). The role of bone-marrow-derived cells in tumor growth, metastasis initiation and progression. Trends Mol. Med..

[10] Murdoch C., Muthana M., Coffelt S.B., Lewis C.E. (2008). The role of myeloid cells in the promotion of tumour angiogenesis. Nat. Rev. Cancer.

[11] Gabrilovich D.I., Ostrand-Rosenberg S., Bronte V. (2012). Coordinated regulation of myeloid cells by tumours. Nat. Rev. Immunol..

[12] Kitamura T., Qian B.Z., Pollard J.W. (2015). Immune cell promotion of metastasis. Nat. Rev. Immunol..

[13] Gabrilovich D.I., Nagaraj S. (2009). Myeloid-derived suppressor cells as regulators of the immune system. Nat. Rev. Immunol..

[14] Talmadge J.E., Gabrilovich D.I. (2013). History of myeloid-derived suppressor cells. Nat. Rev. Cancer.

[15] Fridlender Z.G., Sun J., Kim S., Kapoor V., Cheng G., Ling L., Worthen G.S., Albelda S.M. (2009). Polarization of tumor-associated neutrophil phenotype by TGF-β: “N1” *versus* “N2” TAN. Cancer Cell.

[16] Mantovani A., Cassatella M.A., Costantini C., Jaillon S. (2011). Neutrophils in the activation and regulation of innate and adaptive immunity. Nat. Rev. Immunol..

[17] Templeton A.J., McNamara M.G., Šeruga B., Vera-Badillo F.E., Aneja P., Ocaña A., Leibowitz-Amit R., Sonpavde G., Knox J.J., Tran B. (2014). Prognostic role of neutrophil-to-lymphocyte ratio in solid tumors: A systematic review and meta-analysis. J. Natl. Cancer Inst..

[18] Albini A., Sporn M.B. (2007). The tumor microenvironment as a target for chemoprevention. Nat. Rev. Cancer.

[19] Wang D., Wang H., Brown J., Daikoku T., Ning W., Shi Q., Richmond A., Strieter R., Dey S.K., DuBois R.N. (2006). CXCL1 induced by prostaglandin E2 promotes angiogenesis in colorectal cancer. J. Exp. Med..

[20] Yamamoto M., Kikuchi H., Ohta M., Kawabata T., Hiramatsu Y., Kondo K., Baba M., Kamiya K., Tanaka T., Kitagawa M. (2008). TSU68 prevents liver metastasis of colon cancer xenografts by modulating the premetastatic niche. Cancer Res..

[21] Katoh H., Wang D., Daikoku T., Sun H., Dey S.K., DuBois R.N. (2013). CXCR2-expressing myeloid-derived suppressor cells are essential to promote colitis-associated tumorigenesis. Cancer Cell.

[22] Kawada K., Hosogi H., Sonoshita M., Sakashita H., Manabe T., Shimahara Y., Sakai Y., Takabayashi A., Oshima M., Taketo M.M. (2007). Chemokine receptor CXCR3 promotes colon cancer metastasis to lymph nodes. Oncogene.

[23] Murakami T., Kawada K., Iwamoto M., Akagami M., Hida K., Nakanishi Y., Kanda K., Kawada M., Seno H., Taketo M.M. (2013). The role of CXCR3 and CXCR4 in colorectal cancer metastasis. Int. J. Cancer.

[24] Musha H., Ohtani H., Mizoi T., Kinouchi M., Nakayama T., Shiiba K., Miyagawa K., Nagura H., Yoshie O., Ssaki I. (2005). Selective infiltration of CCR5^+^ CXCR3^+^ T lymphocytes in human colorectal carcinoma. Int. J. Cancer.

[25] Kim J., Takeuchi H., Lam S.T., Turner R.R., Wang H.J., Kuo C., Foshag L., Bilchik A.J., Hoon D.S. (2005). Chemokine receptor CXCR4 expression in colorectal cancer patients increases the risk for recurrence and for poor survival. J. Clin. Oncol..

[26] Ottaiano A., Franco R., Aiello Talamanca A., Liguori G., Tatangelo F., Delrio P., Nasti G., Barletta E., Facchini G., Daniele B. (2006). Overexpression of both CXC chemokine receptor 4 and vascular endothelial growth factor proteins predicts early distant relapse in stage II-III colorectal cancer patients. Clin. Cancer Res..

[27] Schimanski C.C., Schwald S., Simiantonaki N., Jayasinghe C., Gönner U., Wilsberg V., Junginger T., Berger M.R., Galle P.R., Moehler M. (2005). Effect of chemokine receptors CXCR4 and CCR7 on the metastasis behavior of human colorectal cancer. Clin. Cancer Res..

[28] Yopp A.C., Shia J., Butte J.M., Allen P.J., Fong Y., Jarnagin W.R., DeMatteo R.P., D’Angelica M.I. (2012). CXCR4 expression predicts patient outcome and recurrence patterns after hepatic resection for colorectal liver metastases. Ann. Surg. Oncol..

[29] Matsusue R., Kubo H., Hisamori S., Okoshi K., Takagi H., Hida K., Nakano K., Itami A., Kawada K., Nagayama S. (2009). Hepatic stellate cells promote liver metastasis of colon cancer cells by the action of SDF-1/CXCR4 axis. Ann. Surg. Oncol..

[30] Gassmann P., Haier J., Schlüter K., Domikowsky B., Wendel C., Wiesner U., Kubitza R., Engers R., Schneider S.W., Homey B. (2009). CXCR4 regulates the early extravasation of metastatic tumor cells *in vivo*. Neoplasia.

[31] Akishima-Fukasawa Y., Nakanishi Y., Ino Y., Moriya Y., Kanai Y., Hirohashi S. (2009). Prognostic significance of CXCL12 expression in patients with colorectal carcinoma. Am. J. Clin. Pathol..

[32] Bailey C., Negus R., Morris A., Ziprin P., Goldin R., Allavena P., Peck D., Darzi A. (2007). Chemokine expression is associated with the accumulation of tumor associated macrophages (TAMs) and progression in human colorectal cancer. Clin. Exp. Metastasis.

[33] Hu H., Sun L., Guo C., Liu Q., Zhou Z., Peng L., Pan J., Yu L., Lou J., Yang Z. (2009). Tumor cell-microenvironment interaction models coupled with clinical validation reveal CCL2 and SNCG as two predictors of colorectal cancer hepatic metastasis. Clin. Cancer Res..

[34] Zhao L., Lim S.Y., Gordon-Weeks A.N., Tapmeier T.T., Im J.H., Cao Y., Beech J., Allen D., Smart S., Muschel R.J. (2013). Recruitment of a myeloid cell subset (CD11b/Gr1^mid^) via CCL2/CCR2 promotes the development of colorectal cancer liver metastasis. Hepatology.

[35] Chun E., Lavoie S., Michaud M., Gallini C.A., Kim J., Soucy G., Odze R., Glickman J.N., Garrett W.S. (2015). CCL2 Promotes Colorectal Carcinogenesis by Enhancing Polymorphonuclear Myeloid-Derived Suppressor Cell Population and Function. Cell Rep..

[36] Wolf M.J., Hoos A., Bauer J., Boettcher S., Knust M., Weber A., Simonavicius N., Schneider C., Lang M., Stürzl M. (2012). Endothelial CCR2 signaling induced by colon carcinoma cells enables extravasation via the JAK2-Stat5 and p38MAPK pathway. Cancer Cell.

[37] Chang L.Y., Lin Y.C., Mahalingam J., Huang C.T., Chen T.W., Kang C.W., Peng H.M., Chu Y.Y., Chiang J.M., Dutta A. (2012). Tumor-derived chemokine CCL5 enhances TGF-β-mediated killing of CD8^+^ T cells in colon cancer by T-regulatory cells. Cancer Res..

[38] Kitamura T., Kometani K., Hashida H., Matsunaga A., Miyoshi H., Hosogi H., Aoki M., Oshima M., Hattori M., Takabayashi A. (2007). SMAD4-deficient intestinal tumors recruit CCR1^+^ myeloid cells that promote invasion. Nat. Genet..

[39] Kitamura T., Fujishita T., Loestcher P., Revesz L., Hashida H., Kizaka-Kondoh S., Aoki M., Taketo M.M. (2010). Inactivation of Chemokine (C-C motif) receptor 1 (CCR1) suppresses colon cancer liver metastasis by blocking accumulation of immature myeloid cells in a mouse model. Proc. Natl. Acad. Sci. USA.

[40] Itatani Y., Kawada K., Fujishita T., Kakizaki F., Hirai H., Matsumoto T., Iwamoto M., Inamoto S., Hatano E., Hasegawa S. (2013). Loss of SMAD4 from colorectal cancer cells promotes CCL15 expression to recruit CCR1^+^ myeloid cells and facilitate liver metastasis. Gastroenterology.

[41] Hirai H., Fujishita T., Kurimoto K., Miyachi H., Kitano S., Inamoto S., Itatani Y., Saitou M., Maekawa T., Taketo M.M. (2014). CCR1-mediated accumulation of myeloid cells in the liver microenvironment promoting mouse colon cancer metastasis. Clin. Exp. Metastasis.

[42] Inamoto S., Itatani Y., Yamamoto T., Minamiguchi S., Hirai H., Iwamoto M., Hasegawa S., Taketo M.M., Sakai Y., Kawada K. (2016). Loss of SMAD4 promotes colorectal cancer progression by accumulation of myeloid-derived suppressor cells through CCL15–CCR1 chemokine axis. Clin. Cancer Res..

[43] Liu J., Zhang N., Li Q., Zhang W., Ke F., Leng Q., Wang H., Chen J., Wang H. (2011). Tumor-associated macrophages recruit CCR6^+^ regulatory T cells and promote the development of colorectal cancer via enhancing CCL20 production in mice. PLoS ONE.

[44] Geginat J., Lanzavecchia A., Sallusto F. (2003). Proliferation and differentiation potential of human CD8^+^ memory T-cell subsets in response to antigen or homeostatic cytokines. Blood.

[45] Wong J.L., Muthuswamy R., Bartlett D.L., Kalinski P. (2013). IL-18-based combinatorial adjuvants promote the intranodal production of CCL19 by NK cells and dendritic cells of cancer patients. Oncoimmunology.

[46] Zheng J., Yang M., Shao J., Miao Y., Han J., Du J. (2013). Chemokine receptor CX3CR1 contributes to macrophage survival in tumor metastasis. Mol. Cancer.

[47] Kojima Y., Acar A., Eaton E.N., Mellody K.T., Scheel C., Ben-Porath I., Onder T.T., Wang Z.C., Richardson A.L., Weinberg R.A. (2010). Autocrine TGF-β and stromal cell-derived factor-1 (SDF-1) signaling drives the evolution of tumor-promoting mammary stromal myofibroblasts. Proc. Natl. Acad. Sci. USA.

[48] Romain B., Hachet-Haas M., Rohr S., Brigand C., Galzi J.L., Gaub M.P., Pencreach E., Guenot D. (2014). Hypoxia differentially regulated CXCR4 and CXCR7 signaling in colon cancer. Mol. Cancer.

[49] Wendt M.K., Johanesen P.A., Kang-Decker N., Binion D.G., Shah V., Dwinell M.B. (2006). Silencing of epithelial CXCL12 expression by DNA hypermethylation promotes colonic carcinoma metastasis. Oncogene.

[50] Brand S., Dambacher J., Beigel F., Olszak T., Diebold J., Otte J.M., Göke B., Eichhorst S.T. (2005). CXCR4 and CXCL12 are inversely expressed in colorectal cancer cells and modulate cancer cell migration, invasion and MMP-9 activation. Exp. Cell Res..

[51] Ma L., Qiao H., He C., Yang Q., Cheung C.H., Kanwar J.R., Sun X. (2012). Modulating the interaction of CXCR4 and CXCL12 by low-molecular-weight heparin inhibits hepatic metastasis of colon cancer. Investig. New Drugs.

[52] Duda D.G., Kozin S.V., Kirkpatrick N.D., Xu L., Fukumura D., Jain R.K. (2011). CXCL12 (SDF1α)-CXCR4/CXCR7 pathway inhibition: An emerging sensitizer for anticancer therapies?. Clin. Cancer Res..

[53] Du R., Lu K.V., Petritsch C., Liu P., Ganss R., Passegué E., Song H., Vandenberg S., Johnson R.S., Werb Z. (2008). HIF1α induces the recruitment of bone marrow-derived vascular modulatory cells to regulate tumor angiogenesis and invasion. Cancer Cell.

[54] Hiratsuka S., Duda D.G., Huang Y., Goel S., Sugiyama T., Nagasawa T., Fukumura D., Jain R.K. (2011). C-X-C receptor type 4 promotes metastasis by activating p38 mitogen-activated protein kinase in myeloid differentiation antigen (Gr-1)-positive cells. Proc. Natl. Acad. Sci. USA.

[55] Kaplan R.N., Riba R.D., Zacharoulis S., Bramley A.H., Vincent L., Costa C., MacDonald D.D., Jin D.K., Shido K., Kerns S.A. (2005). VEGFR1-positive haematopoietic bone marrow progenitors initiate the pre-metastatic niche. Nature.

[56] Zhong C., Wang J., Li B., Xiang H., Ultsch M., Coons M., Wong T., Chiang N.Y., Clark S., Clark R. (2013). Development and preclinical characterization of a humanized antibody targeting CXCL12. Clin. Cancer Res..

[57] Kawada K., Sonoshita M., Sakashita H., Takabayashi A., Yamaoka Y., Manabe T., Inaba K., Minato N., Oshima M., Taketo M.M. (2004). Pivotal role of CXCR3 in melanoma cell metastasis to lymph nodes. Cancer Res..

[58] Kawada K., Taketo M.M. (2011). Significance and mechanism of lymph node metastasis in cancer progression. Cancer Res..

[59] Zipin-Roitman A., Meshel T., Sagi-Assif O., Shalmon B., Avivi C., Pfeffer R.M., Witz I.P., Ben-Baruch A. (2007). CXCL10 promotes invasion-related properties in human colorectal carcinoma cells. Cancer Res..

[60] Longo-Imedio M.I., Longo N., Trevino I., Lazaro P., Sanchez-Mateos P. (2005). Clinical significance of CXCR3 and CXCR4 expression in primary melanoma. Int. J. Cancer.

[61] Ma X., Norsworthy K., Kundu N., Rodgers W.H., Gimotty P.A., Goloubeva O., Lipsky M., Li Y., Holt D., Fulton A. (2009). CXCR3 expression is associated with poor survival in breast cancer and promotes metastasis in a murine model. Mol. Cancer Ther..

[62] Cambien B., Karimdjee B.F., Richard-Fiardo P., Bziouech H., Barthel R., Millet M.A., Martini V., Birnbaum D., Scoazec J.Y., Abello J. (2009). Organ-specific inhibition of metastatic colon carcinoma by CXCR3 antagonism. Br. J. Cancer.

[63] Walser T.C., Rifat S., Ma X., Kundu N., Ward C., Goloubeva O., Johnson M.G., Mediana J.C., Colloins T.L., Fulton A.M. (2006). Antagonism of CXCR3 inhibits lung metastasis in a murine model of metastatic breast cancer. Cancer Res..

[64] Zhu G., Yan H.H., Pang Y., Jian J., Achyut B.R., Liang X., Weiss J.M., Wiltrout R.H., Hollander M.C., Yang L. (2015). CXCR3 as a molecular target in breast cancer metastasis: Inhibition of tumor cell migration and promotion of host anti-tumor immunity. Oncotarget.

[65] Jiang Z., Xu Y., Cai S. (2010). CXCL10 expression and prognostic significance in stage II and III colorectal cancer. Mol. Biol. Rep..

[66] Agesen T.H., Sveen A., Merok M.A., Lind G.E., Nesbakken A., Skotheim R.I., Lothe R.A. (2012). ColoGuideEx: A robust gene classifier specific for stage II colorectal cancer prognosis. Gut.

[67] Wightman S.C., Uppal A., Pitroda S.P., Ganai S., Burnette B., Stack M., Oshima G., Khan S., Huang X., Posner M.C. (2015). Oncogenic CXCL10 signaling drives metastasis development and poor clinical outcome. Br. J. Cancer.

[68] Toiyama Y., Fujikawa H., Kawamura M., Matsushita K., Saigusa S., Tanaka K., Inoue Y., Uchida K., Mohri Y., Kusunoki M. (2012). Evaluation of CXCL10 as a novel serum marker for predicting liver metastasis and prognosis in colorectal cancer. Int. J. Oncol..

[69] Sonoshita M., Takaku K., Sasaki N., Sugimoto Y., Ushikubi F., Narumiya S., Oshima M., Taketo M.M. (2001). Acceleration of intestinal polyposis through prostaglandin receptor EP2 in *Apc^Δ716^* knockout mice. Nat. Med..

[70] Rubie C., Frick V.O., Wagner M., Schuld J., Graber S., Brittner B., Bohle R.M., Schilling M.K. (2008). ELR^+^ CXC chemokine expression in benign and malignant colorectal conditions. BMC Cancer.

[71] Bandapalli O.R., Ehrmann F., Ehemann V., Gaida M., Macher-Goeppinger S., Wente M., Schimacher P., Brand K. (2012). Down-regulation of CXCL1 inhibits tumor growth in colorectal liver metastasis. Cytokine.

[72] Minn A.J., Gupta G.P., Siegel P.M., Bos P.D., Shu W., Giri D.D., Viale A., Olshen A.B., Gerald W.L., Masagué J. (2005). Genes that mediate breast cancer metastasis to lung. Nature.

[73] Yang L., Huang J., Ren X., Gorska A.E., Chytil A., Aakre M., Carbone D.P., Matrisian L.M., Richmond A., Lin P.C. (2008). Abrogation of TGFβ signaling in mammary carcinomas recruits Gr-1^+^CD11b^+^ myeloid cells that promote metastasis. Cancer Cell.

[74] Novitskiy S.V., Pickup M.W., Gorska A.E., Owens P., Chytil A., Aakre M., Wu H., Shyr Y., Moses H.L. (2011). TGF-β receptor II loss promotes mammary carcinoma progression by Th17 dependent mechanisms. Cancer Discov..

[75] Lesokhin A.M., Hohl T.M., Kitano S., Cortez C., Hirschhorn-Cymerman D., Avogadri F., Rizzuto G.A., Lazarus J.J., Pamer E.G., Houghton A.N. (2012). Monocytic CCR2^+^ myeloid-derived suppressor cells promote immune escape by limiting activated CD8 T-cell infiltration into the tumor microenvironment. Cancer Res..

[76] Zhang B., Wang Z., Wu L., Zhang M., Li W., Ding J., Zhu J., Wei H., Zhao K. (2013). Circulating and tumor-infiltrating myeloid-derived suppressor cells in patients with colorectal carcinoma. PLoS ONE.

[77] Highfill S.L., Cui Y., Giles A.J., Smith J.P., Zhang H., Morse E., Kaplan R.N., Mackall C.L. (2014). Disruption of CXCR2-mediated MDSC tumor trafficking enhances anti-PD1 efficacy. Sci. Transl. Med..

[78] Yang S.K., Eckmann L., Panja A., Kagnoff M.F. (1997). Differential and regulated epression of C-X-C, C-C, and C-chemokines by human colon epithelial cells. Gastroenterology.

[79] Mizukami Y., Jo W.S., Duerr E.M., Gala M., Li J., Zhang X., Zimmer M.A., Iliopoulos O., Zukerberg L.R., Kohgo Y. (2005). Induction of interleukin-8 preserves the angiogenic response in HIF-1-α-deficient colon cancer cells. Nat. Med..

[80] Ning Y., Manegold P.C., Hong Y.K., Zhang W., Pohl A., Lurje G., Winder T., Yang D., LaBonte M.J., Wilson P.M. (2011). Interkeukin-8 is associated with proliferation, migration, angiogenesis and chemosensitivity *in vitro* and *in vivo* in colon cancer cell line models. Int. J. Cancer.

[81] Asfaha S., Dubeykovskiy A.N., Tomita H., Yang X., Stokes S., Shibata W., Friedman R.A., Ariyama H., Dubeykovskaya Z.A., Muthupalani S. (2013). Mice that express human interleukin-8 have increased mobilization of immature myeloid cells, which exacerbates inflammation and accelerates colon carcinogenesis. Gastroenterology.

[82] Lee Y.S., Choi I., Ning Y., Kim N.Y., Khatchadourian V., Yang D., Chung H.K., Choi D., LaBonte M.J., Ladner R.D. (2012). Interleukin-8 and its receptor CXCR2 in the tumour microenvironment promote colon cancer growth, progression and metastasis. Br. J. Cancer.

[83] Varney M.L., Singh S., Li A., Mayer-Ezell R., Bond R., Singh R.K. (2011). Small molecule antagonists for CXCR2 and CXCR1 inhibit human colon cancer liver metastases. Cancer Lett..

[84] Salovaara R., Roth S., Loukola A., Launonen V., Sistonen P., Avizienyte E., Kristo P., Järvinen H., Souchelnytskyi S., Sarlomo-Rikala M. (2002). Frequent loss of SMAD4/DPC4 protein in colorectal cancers. Gut.

[85] Losi L., Bouzourene H., Benhattar J. (2007). Loss of Smad4 expression predicts liver metastasis in human colorectal cancer. Oncol. Rep..

[86] Kawakami M., Yamaguchi T., Takahashi K., Matsumoto H., Yasutome M., Horiguchi S., Hayashi Y., Funata N., Mori T. (2010). Assessment of SMAD4, p53, and Ki-67 alterations as a predictor of liver metastasis in human colorectal cancer. Surg. Today.

[87] Takaku K., Oshima M., Miyoshi H., Matsui M., Seldin M.F., Taketo M.M. (1998). Intestinal tumorigenesis in compound mutant mice of both Dpc4 (Smad4) and Apc genes. Cell.

[88] Rodero M.P., Auvynet C., Poupel L., Combadière B., Combadière C. (2013). Control of both myeloid cell infiltration and angiogenesis by CCR1 promotes liver cancer metastasis development in mice. Neoplasia.

[89] Kitamura T., Qian B.Z., Soong D., Cassetta L., Noy R., Sugano G., Kato Y., Li J., Pollard J.W. (2015). CCL2-induced chemokine cascade promotes breast cancer metastasis by enhancing retention of metastasis-associated macrophages. J. Exp. Med..

[90] Gladue R.P., Brown M.F., Zwillich S.H. (2010). CCR1 antagonists: What have we learned from clinical trials. Curr. Top. Med. Chem..

[91] Szekanecz Z., Koch A.E. (2016). Successes and failures of chemokine-pathway targeting in rheumatoid arthritis. Nat. Rev. Rheumatol..

[92] Nakanishi Y., Nakatsuji M., Seno H., Ishizu S., Akitake-Kawano R., Kanda K., Ueo T., Komekado H., Kawada M., Minami M. (2011). COX-2 inhibition alters the phenotype of tumor-associated macrophages from M2 to M1 in *Apc^Min/+^* mouse polyps. Carcinogenesis.

[93] Tanaka S., Tatsuguchi A., Futagami S., Gudis K., Wada K., Seo T., Mitsui K., Yonezawa M., Nagata K., Fujimori S. (2006). Monocyte chemoattractant protein 1 and macrophage cyclooxygenase 2 expression in colonic adenoma. Gut.

[94] Qian B.Z., Li J., Zhang H., Kitamura T., Zhang J., Campion L.R., Kaiser E.A., Snyder L.A., Pollard J.W. (2011). CCL2 recruits inflammatory monocytes to facilitate breast-tumour metastasis. Nature.

[95] Zhang J., Lu Y., Pienta K.J. (2010). Multiple roles of chemokine (C-C motif) ligand 2 in promoting prostate cancer growth. J. Natl. Cancer Inst..

[96] McClellan J.L., Davis J.M., Steiner J.L., Enos R.T., Jung S.H., Carson J.A., Pena M.M., Carnevale K.A., Berger F.G., Murphy E.A. (2012). Linking tumor-associated macrophages, inflammation, and intestinal tumorigenesis: Role of MCP-1. Am. J. Physiol. Gastrointest. Liver Physiol..

[97] Popivanova B.K., Kostadinova F.I., Furuichi K., Shamekh M.M., Kondo T., Wada T., Egashira K., Mukaida N. (2009). Blockade of a chemokine, CCL2, reduces chronic colitis-associated carcinogenesis in mice. Cancer Res..

[98] Marazioti A., Kairi C.A., Spella M., Giannou A.D., Magkouta S., Giopanou I., Papaleonidopoulos V., Kalomenidis I., Snyder L.A., Kardamakis D. (2013). Beneficial impact of CCL2 and CCL12 neutralization on experimental malignant pleural effusion. PLoS ONE.

[99] Sanford D.E., Belt B.A., Panni R.Z., Mayer A., Deshpande A.D., Carpenter D., Mitchem J.B., Plambeck-Suess S.M., Worley L.A., Goetz B.D. (2013). Inflammatory monocyte mobilization decreases patient survival in pancreatic cancer: A role for targeting the CCL2/CCR2 axis. Clin. Cancer Res..

[100] Bonapace L., Coissieux M.M., Wyckoff J., Mertz K.D., Varga Z., Junt T., Bentires-Alj M. (2014). Cessation of CCL2 inhibition accelerates breast cancer metastasis by promoting angiogenesis. Nature.

[101] Pittenger M.F. (1999). Multilineage potential of adult human mesenchymeal stem cells. Science.

[102] Torsvik A., Bjerkvig R. (2013). Mesenchymal stem cell signaling in cancer progression. Cancer Treat. Rev..

[103] Chaturvedi P., Gilkes D.M., Wong C.C.L., Luo W., Zhang H., Wei H., Takano N., Schito L., Levchenko A., Semenza G.L. (2013). Hypoxia-inducible factor-dependent breast cancer-mesenchymal stem cell bidirectional signaling promotes metastasis. J. Clin. Investig..

[104] Galon J., Costes A., Sanchez-Cabo F., Kirilovsky A., Mlecnik B., Lagorce-Pagès C., Tosolini M., Camus M., Berger A., Wind P. (2006). Type, density, and location of immune cells within human colorectal tumors predict clinical outcome. Science.

[105] Pagès F., Berger A., Camus M., Sanchez-Cabo F., Costes A., Molidor R., Mlecnik B., Kirilovsky A., Nilsson M., Damotte D. (2005). Effector memory T cells, early metastasis, and survival in colorectal cancer. N. Engl. J. Med..

[106] Naito Y., Saito K., Shiiba K., Ohuchi A., Saigenji K., Nagura H., Ohtani H. (1998). CD8^+^ T cells infiltrated within cancer cell nests as a prognostic factor in human colorectal cancer. Cancer Res..

[107] Cambien B., Richard-Fiardo P., Karimdjee B.F., Martini V., Ferrua B., Pitard B., Schmid-Antomarchi H., Schimid-Alliana A. (2011). CCL5 neutralization restricts cancer growth and potentiates the targeting of PDGFRβ in colorectal carcinoma. PLoS ONE.

[108] Tosolini M., Kirilovsky A., Mlecnik B., Fredriksen T., Mauger S., Bindea G., Berger A., Bruneval P., Fridman W.H., Pagès F. (2011). Clinical impact of different classes of infiltrating T cytotoxic and helper cells (Th1, Th2, Treg, Th17) in patients with colorectal cancer. Cancer Res..

[109] Chung A.S., Wu X., Zhuang G., Ngu H., Kasman I., Zhang J., Vernes J.M., Jiang Z., Meng Y.G., Peale F.V. (2013). An interleukin-17-mediated paracrine network promotes tumor resistance to anti-angiogenic therapy. Nat. Med..

[110] Zou W. (2006). Regulatory T cells, tumour immunity and immunotherapy. Nat. Rev. Immunol..

[111] Yamazaki T., Yang X.O., Chung Y., Fukunaga A., Nurieva R., Pappu B., Martin-Orozco N., Kang H.S., Ma L., Panopoulos A.D. (2008). CCR6 Regulates the Migration of Inflammatory and Regulatory T Cells. J. Immunol..

[112] Kryczek I., Wu K., Zhao E., Wei S., Vatan L., Szeliga W., Huang E., Greenson J., Chang A., Rolinski J. (2011). IL-17^+^ regulatory T cells in the microenvironments of chronic inflammation and cancer. J. Immunol..

[113] Ghadjar P., Rubie C., Aebersold D.M., Keilholz U. (2009). The chemokine CCL20 and its receptor CCR6 in human malignancy with focus on colorectal cancer. Int. J. Cancer.

[114] Frick V.O., Rubie C., Kölsch K., Wagner M., Ghadjar P., Graeber S., Glanemann M. (2013). CCR6/CCL20 chemokine expression profile in distinct colorectal malignancies. Scand. J. Immunol..

[115] Dwinell M.B., Eckmann L., Leopard J.D., Varki N.M., Kagnoff M.F. (1999). Chemokine receptor expression by human intestinal epithelial cells. Gastroenterology.

[116] Ghadjar P., Coupland S.E., Na I.K., Noutsias M., Letsch A., Stroux A., Bauer S., Buhr H.J., Thiel E., Scheibenbogen C. (2006). Chemokine receptor CCR6 expression level and liver metastases in colorectal cancer. J. Clin. Oncol..

[117] Iwata T., Tanaka K., Inoue Y., Toiyama Y., Hiro J., Fujikawa H., Okugawa Y., Uchida K., Mohri Y., Kusunoki M. (2013). Macrophage inflammatory protein-3 alpha (MIP-3α) is a novel serum prognostic marker in patients with colorectal cancer. J. Surg. Oncol..

[118] Nandi B., Pai C., Huang Q., Prabhala R.H., Munshi N.C., Gold J.S. (2014). CCR6, the sole receptor for the chemokine CCL20, promotes spontaneous intestinal tumorigenesis. PLoS ONE.

[119] Palucka K., Banchereau J. (2012). Cancer immunotherapy via dendritic cells. Nat. Rev. Cancer.

[120] Lu J., Zhao J., Feng H., Wang P., Zhang Z., Zong Y., Ma J., Zheng M., Lu A. (2014). Antitumor efficacy of CC motif chemokine ligand 19 in colorectal cancer. Dig. Dis. Sci..

[121] Mumtaz M., Wagsater D., Lofgren S., Hugander A., Zar N., Dimberg J. (2009). Decreased expression of the chemokine CCL21 in human colorectal adenocarcinomas. Oncol. Rep..

[122] Correale P., Rotundo M.S., Botta C., del Vecchio M.T., Ginanneschi C., Licchetta A., Conca R., Apollinari S., de Luca F., Tassone P. (2012). Tumor infiltration by T lymphocytes expressing chemokine receptor 7 (CCR7) is predictive of favorable outcome in patients with advanced colorectal carcinoma. Clin. Cancer Res..

[123] Günther K., Leier J., Henning G., Dimmler A., Weiβbach R., Hohenberger W., Förster R. (2005). Prediction of lymph node metastasis in colorectal carcinoma by expressionof chemokine receptor CCR7. Int. J. Cancer.

[124] Li J., Sun R., Tao K., Wang G. (2011). The CCL21/CCR7 pathway plays a key role in human colon cancer metastasis through regulation of matrix metalloproteinase-9. Dig. Liver Dis..

[125] Cheadle E.J., Riyad K., Subar D., Rothwell D.G., Ashton G., Batha H., Sherlock D.J., Hawkins R.E., Gilham D.E. (2007). Eotaxin-2 and colorectal cancer: A potential target for immune therapy. Clin. Cancer Res..

[126] Shinagawa K., Kitadai Y., Tanaka M., Sumida T., Kodama M., Higashi Y., Tanaka S., Yasui W., Chayama K. (2010). Mesenchymal stem cells enhance growth and metastasis of colon cancer. Int. J. Cancer.

[127] Huang Y.F., Chen M.J., Wu M.H., Hung S.C. (2013). The use of hypoxic cultured mesenchymal stem cell for oncolytic virus therapy. Cancer Gene Ther..

[128] Tardáguila M., Mañes S. (2014). The complex role of chemokines in cancer: The case of the CX3CL1/CX3CR1 axis. Oncology Theory & Practice.

[129] Vitale S., Cambien B., Karimdjee B.F., Barthel R., Staccini P., Luci C., Breittmayer B., Anjuère F., Schmid-Alliana A., Schmid-Antomarchi H. (2007). Tissue-specific differential antitumour effect of molecular forms of fractalkine in a mouse model of metastatic colon cancer. Gut.

[130] Ohta M., Tanaka F., Yamaguchi H., Sadanaga N., Inoue H., Mori M. (2005). The high expression of Fractalkine results in a better prognosis for colorectal cancer patients. Int. J. Oncol..

[131] Le D.T., Uram J.N., Wang H., Barlett B.R., Kemberling H., Eyring A.D., Skora A.D., Luber B.S., Azad N.S., Laheru D. (2015). PD-1 blockade in tumors with mismatch-repair deficiency. N. Eng. J. Med..

[132] Cancer Genome Atlas Network (2012). Comprehensive molecular characterization of human colon and rectal cancer. Nature.

